# Health-related quality of life among patients with type 2 diabetes mellitus in Eastern Province, Saudi Arabia: A cross-sectional study

**DOI:** 10.1371/journal.pone.0227573

**Published:** 2020-01-10

**Authors:** Dhfer Alshayban, Royes Joseph

**Affiliations:** Department of Pharmacy Practice, College of Clinical Pharmacy, Imam Abdulrahman Bin Faisal University, Dammam, Saudi Arabia; Suez Canal University Faculty of Medicine, EGYPT

## Abstract

Diabetes mellitus has reached epidemic levels, and it threatens the economy and health globally and Saudi Arabia in particular. The study assessed health-related quality of life using EuroQol instrument and its predictors among patients with Type 2 diabetes mellitus in Eastern Province, Saudi Arabia. A cross-sectional study was conducted among 378 patients with Type 2 diabetes mellitus from two major health centers in Eastern Province. The study showed moderate health-related quality of life, as reported by the median index score of 0.808 with more than a quarter of patients with severe-extreme health state in some or all domains. Multiple-regression models showed that male gender, high monthly income, having no diabetes-related complications and having random blood glucose level less than 200 mg/dl were prone to have a higher index score compared to the corresponding contrary groups. The study will help in guiding the development of effective intervention programs to improve diabetes-related health-related quality of life among the Saudi population.

## Introduction

Diabetes mellitus (DM) and related complications have reached epidemic levels, and it threatens the economy and health globally. According to the International Diabetes Federation (IDF) reports, 1 in 11 adults aged 20–79 years (425 million adults; 451 million if the age is expanded to 18–99 years) had DM globally in 2017, and 90% of them were with type 2 diabetes mellitus (T2DM) [1,2]. The prevalence and incidence of DM are increasing worldwide, and a rapid progression has been reported in middle- and low-income countries [1]. The new edition of the IDF Diabetes Atlas (8^th^ ed. 2017) reports that approximately 9.2% of adults aged 18–99 years (39.9 million people) had DM in the Middle East and North Africa Region (MENA) in 2017 [1]. It is expected that the number of people with diabetes in the MENA region will be more than double by 2045 [1]. Based on the IDF Diabetes Atlas report, Saudi Arabia is on the top among the MENA countries with the highest age-adjusted DM prevalence of 17.7%, and 4^th^ place in terms of the number of people with diabetes [1]. IDF predicts that approximately one in four adults in Saudi Arabia will have diabetes by 2045 [1]. These estimates indicate that DM has reached epidemic levels, and the chronic condition threatens the global economy and health as it drains national health care budgets and reduces productivity [1,3].

DM is a significant and growing healthcare challenge in Saudi Arabia primarily because of increased physical inactivity, consumption of unhealthy diets, obesity and sedentary lifestyles [4,5]. DM is a major cause of blindness, kidney failure, heart attacks, stroke and lower limb amputation [6]. DM and its complications have contributed tremendously to the burden of mortality and disability worldwide [6]. The Global Burden of Disease Study 2015 identified DM as the ninth major cause of reduced life expectancy and reported that high fasting level of glucose was the third most common global risk factor for disability-adjusted life years in 2015 [7]. WHO reports that diabetes was the seventh leading cause of death in 2016 [3]. According to IDF Diabetes Atlas 2017, an estimated four million deaths were directly caused by DM [1]. In Saudi Arabia, the expected number of death due to DM was 14700 in 2017, and 70% of them were expected to be aged under 60 years [1].

Quality of life (QoL) indicators are solid predictors of an individual’s competence to maintain long-term health, well -being and productivity [8]. Improved QoL has been regarded as a key goal of all healthcare interventions including DM management programs [9]. Previous studies reported that DM and its complications drain a substantial portion of the national healthcare budget in Saudi Arabia [1,10]. Hence, it is important to know the level of health-related QoL (HRQoL) of diabetes patients against the huge spending from the national budget. Identifying factors that are associated with impaired HRQoL may help policymakers to prioritize funding and implement interventions to improve the QoL.

Studies from Saudi Arabia [11–13], other Middle Eastern countries [14–16], and rest of the world [17,18] show that diabetes impairs the QoL of patients, but the level of impairment was not the same across the studies. A recent review indicated that Saudi Arabia’s direct spending on diabetes was almost 14% of the total health expenditure, and the study urged for improving health and HRQoL of diabetes patients in order to reduce the social and personal costs for diabetes care in Saudi Arabia [5]. Apart from three regional level studies (from Makkah and Riyadh regions), a national level study on HRQoL among diabetes patients in Saudi Arabia has not been reported during the past decade. Importantly, any QoL studies among diabetes patients from Eastern Province, the largest region of Saudi Arabia, has not been reported previously. EQ-5D is regarded as one of generic instruments, rather than a disease-specific, that has been used extensively in research recently beside other instruments such as SF-36 [19,20]. Among these instruments, the EQ-5D has the benefit of being able to convert health states into a single index value that can be compared among diseases and used for economic evaluation [21]. Therefore, the present study used the EQ-5D instrument to measure HRQoL in T2DM patients in Eastern Province, Saudi Arabia, and to determine the impact of socio-demographic and clinical factors on HRQoL.

## Methodology

### Study setting and subjects

A cross-sectional study was conducted from November 2017 to April 2018 among 378 T2DM patients. Patients were conveniently recruited from two health centers of the King Fahad Hospital of the University, which is a major tertiary hospital in the Eastern Province, Saudi Arabia. One center is located in the Khobar and Dhahran region, and the other one is located in the Dammam region. Hospital statistics of these health centers and collected demographic data of patients indicated that fair representation of patients from several geographical locations within the Eastern Province. A minimum sample size of 385 was calculated by assuming 50% of patients were adherent to treatments with the absolute precision of 0.05 and 95% confidence level. The 50% was purposively selected so that it provided the largest minimum sample size. Patients with minimum age of 18 years and with T2DM for at least 1 year were considered for this study if they provided a written informed consent. Patients with pregnancy or other medical complications were excluded from the study. The study was approved by the Institutional Review Board and the Ethical Committee at Imam Abdulrahman Bin Faisal University (IRB-2019-05-391).

### Data collection

An Arabic version of the EQ-5D questionnaire was used after obtaining prior permission from the EuroQol Research Foundation [22,23]. The participants were interviewed in Arabic, and their socio-demographic and clinical characteristics were obtained. The EQ-5D questionnaire was filled by the participants.

#### Socio-demographic and clinical characteristics

The data on participants’ gender, age, education status, monthly income, number of diabetes-related complications, current use of anti-diabetic medications (type and number), and random blood glucose level were collected.

#### Assessment of HRQoL

HRQoL was assessed using the EQ-5D-5L [24]. The EQ-5D-5L involves patient self-reporting of their health status in terms of five dimensions: mobility, self-care, usual activities, pain/discomfort, and anxiety/depression. Each dimension has a five-level severity scale (no problems, slight, moderate, severe and extreme) scored from 1 to 5. Five-digit codes for the HRQoL of each patient are obtained from the score digits; there are 3125 possible sets of values, called health states, for EQ-5D-5L. The health states would range from 11111 (perfect health) to 55555 (worst health) and can be converted into a single weighted index score (EQ-5D index) using population preference scores. We used the EQ-5D-5L value set for England to derive the EQ-5D index [21]. Thus, a health state yields index score of between -0.285 and 1: index score 1 represents perfect health, 0 represents a health state equivalent to death and a score of less than 0 represents health states worse than death.

### Statistical analysis

Socio-demographic and disease characteristics of the participants were summarized using descriptive statistics. Percentages and frequencies were used for the categorical variables, while median and interquartile range were calculated for the continuous variables. Association of socio-demographic and clinical factors on HRQoL was assessed using three approaches: 1). Using chi-square test where EQ-5D health states were divided into three categories (perfect health indicates no problem in domains of EQ-5D; slight/moderate indicates problems in some domains but not worse than moderate health in any domains; severe/unable indicates a health status with problems worse than moderate health in some domains). 2). Using a multiple logistic regression with forward selection (likelihood ratio) of predictor variables, where the outcome variable was a binary variable indicating ‘perfect health’ (EQ-5D index = 1.000) or ‘imperfect health’ (EQ-5D index <1.000). 3). Using a multiple linear regression where the dependent variable was the cubic function of EQ-5D index score (the cubic function ensured normally distributed residuals). A p-value less than 0.05 was considered as statistically significant. All analyses were carried out using SPSS Statistics 24.0.

## Results

### Socio-demographic and clinical characteristics of participants

Table 1 presents the socio-demographic and clinical characteristics of participants. Among the 378 participants, half of them were male, 79% were older than 50 years, more than 50% had an education of high school or more, and more than half of them had monthly income of 5000sar or more. Regarding the clinical characteristics of the participants, 78% had diabetes related complications, 61% were on oral anti-diabetic medications only and 70% were on multiple anti-diabetic medications. 48% of participants had random glucose level of 200 mg/dl or more.

**Table 1:** Socio-demographic and clinical characteristics of participants (N = 378).

Variables	n (%^#^)
Gender	
Male	182 (48.1%)
Female	186 (49.2%)
Age	
<50 years	78 (20.6%)
>50 years	298 (78.8%)
Education status	
Primary or lower	176 (46.6%)
High school/Secondary	128 (33.9%)
College graduate	74 (19.6%)
Monthly income (in SAR)	
Less than 5000	162 (42.9%)
5000 to 10000	98 (25.9%)
More than 10000	112 (29.6%)
Number of diabetes related complications	
Nil	84 (22.2%)
One	120 (31.7%)
More than one	174 (46.0%)
Type of anti-diabetic medication	
Insulin injection or combination	144 (38.1%)
Oral medication only	230 (60.8%)
Number of anti-diabetic medications using	
One medication	114 (30.2%)
Two medications	166 (43.9%)
Three or more medications	82 (21.7%)
Random blood glucose level	
less than 200	196 (51.9%)
200 to 299	134 (35.4%)
More than 300	48 (12.7%)

^#^few observations were missing on some variables, but % was calculated based on 378.

### Health-related quality of life

**Fig 1:**
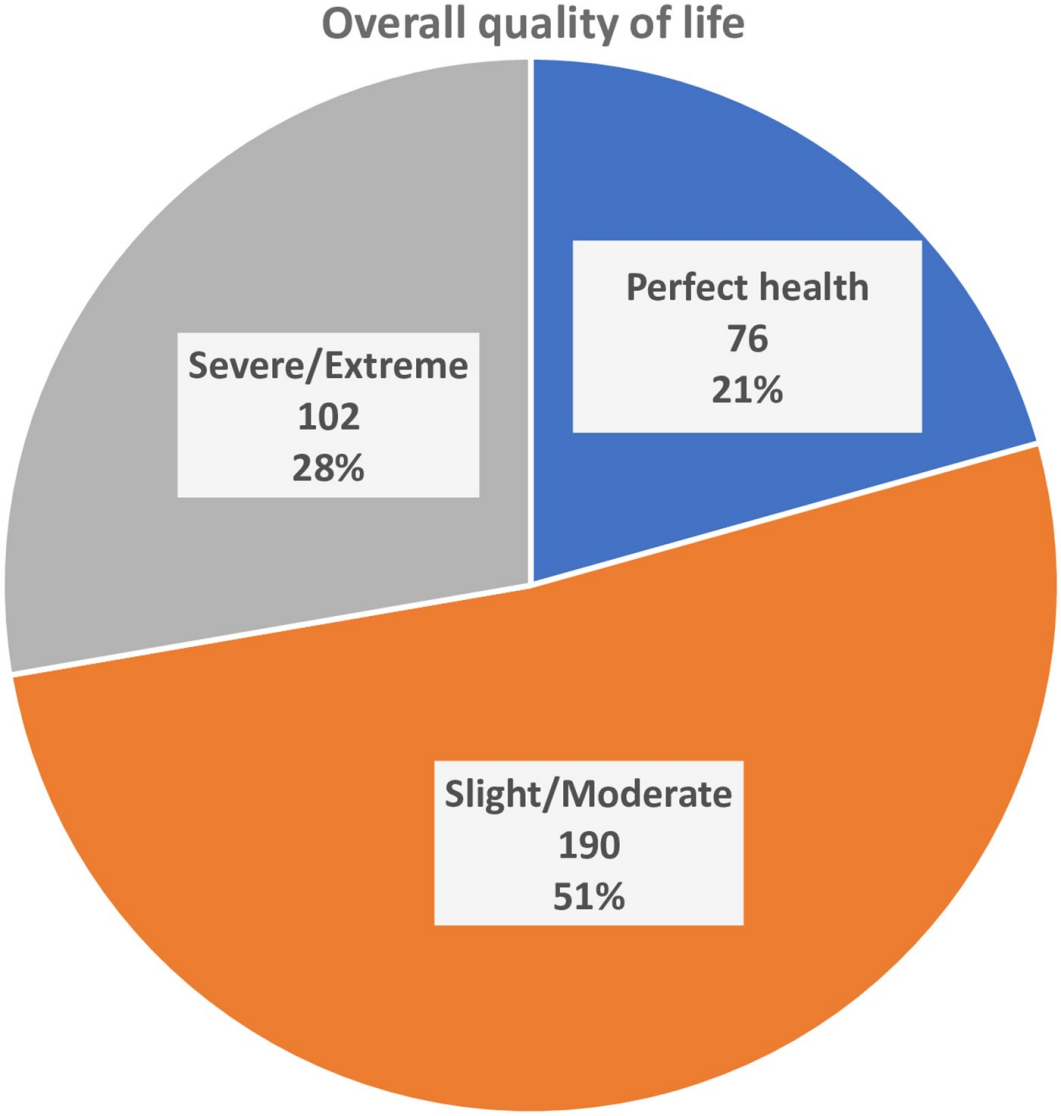
Health-related quality of life measured using EQ-5D-5L scale.

**Fig 2:**
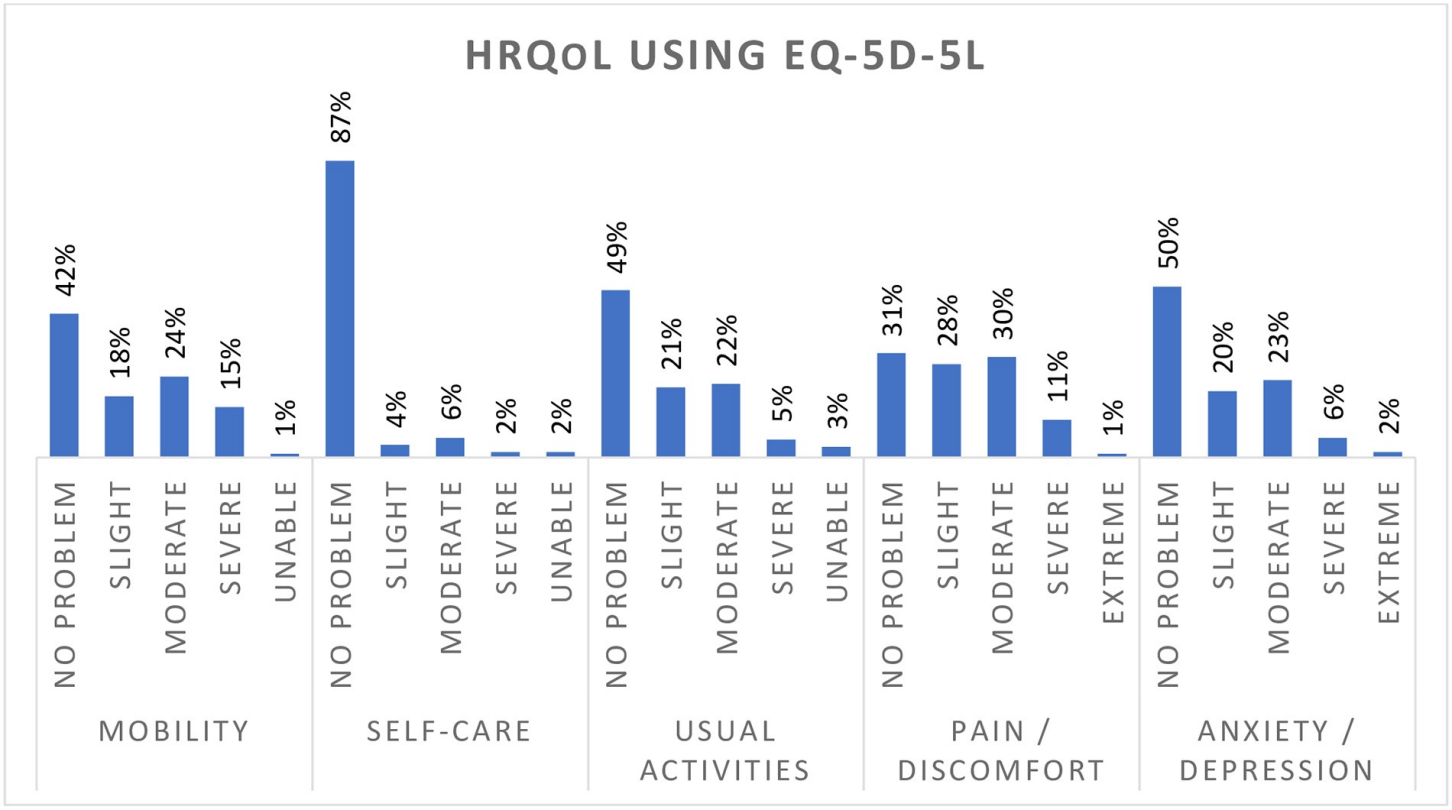
Overall health-related quality of life. Perfect health indicates no problem in domains of EQ-5D; Slight/moderate indicates problems in some domains but not worse than moderate health in any domains; Severe/unable indicates a health status with problems worse than moderate health in some domains. 10 participants did not respond to some domains.

Fig 1 shows the patients’ response over five levels in each of the five domains of EQ-5D. Among the respondents, 88%, 51%, 50%, 43% and 31% were agreed as having no problem in terms of self-care, anxiety or depression, usual activities, mobility, and pain or discomfort respectively. In combined, as shown in Fig 2, one-fifth (76/368) of patients did not have a problem in any domains of EQ-5D (called perfect health state); half of the participants (190/368) reported as having problems in some domains but not worse than moderate health in any domains (called slight-moderate health state); and the remaining 28% (162/368) reported as having problems worse than moderate health in some domains (called severe-extreme health state). The median (interquartile range) EQ-5D index was 0.808 (0.647–0.937).

Table 2 presents the association of socio-demographic and clinical factors on the level of HRQoL based on univariate analyses. It reports the percentage of participants with perfect health, slight-moderate health and severe-moderate health states within each level of factors of interest. A higher percentage of participants with perfect health state and a lower percentage of participants with severe-extreme health state were reported among male gender (p-value<0.001), patients aged 50 years or lower (p-value = 0.026), college graduates (p-value<0.001), patients with monthly income greater than 5000sar (p-value<0.001), patients having no diabetes-related complications (p-value<0.001), patients taking only oral anti-diabetic medication (p-value = 0.014), and patients with RBG less than 200 mg/dl (p-value<0.001) compared to that in the corresponding contrary groups.

**Table 2:** Overall health related quality of life (Univariate analysis).

Factors	Overall health status			
	Perfect health	Slight/Moderate	Severe/Extreme	p-value^a^
Gender				
Male	64 (35.6%)	92 (51.1%)	24 (13.3%)	<0.001
Female	12 (6.7%)	94 (52.8%)	72 (40.4%)	
Age				
50 years	14 (18.9%)	48 (64.9%)	12 (16.2%)	0.026
50 years	62 (21.2%)	142 (48.6%)	88 (30.1%)	
Education status				
Primary or lower	24 (14.1%)	78 (45.9%)	68 (40%)	<0.001
High/Secondary	26 (20.6%)	76 (60.3%)	24 (19%)	
College graduate	26 (36.1%)	36 (50%)	10 (13.9%)	
Monthly income (in SAR)				
Less than 5000	18 (11.4%)	74 (46.8%)	66 (41.8%)	<0.001
5000 to 10000	26 (27.7%)	60 (63.8%)	8 (8.5%)	
More than 10000	32 (29.1%)	54 (49.1%)	24 (21.8%)	
Number of diabetes related complications				
Nil	24 (29.3%)	48 (58.5%)	10 (12.2%)	<0.001
One	36 (30%)	58 (48.3%)	26 (21.7%)	
More than one	16 (9.6%)	84 (50.6%)	66 (39.8%)	
Type of anti-diabetic medication				
Insulin injection or combination	20 (14.1%)	74 (52.1%)	48 (33.8%)	0.014
Only oral medication	56 (25.2%)	114 (51.4%)	52 (23.4%)	
Number of anti-diabetic medications using				
One medication	26 (23.6%)	64 (58.2%)	20 (18.2%)	0.101
Two medications	34 (21.3%)	76 (47.5%)	50 (31.3%)	
Three or more	16 (19.5%)	38 (46.3%)	28 (34.1%)	
Random blood glucose level				
less than 200	64 (33.7%)	94 (49.5%)	32 (16.8%)	<0.001
200 to 299	10 (7.6%)	72 (54.5%)	50 (37.9%)	
More than 300	2 (4.3%)	24 (52.2%)	20 (43.5%)	

^a^Chi-square test was used

### Predictors of severe/extreme health state: Binomial modeling

Results from a multiple logistic regression, where the outcome variable was a binary variable indicating ‘severe/extreme health state in some or all domain’ or ‘not’, is presented in Table 3. Table 3 reports the adjusted odds ratio and its 95% confidence interval for the considered factors. The adjusted odds ratio for gender indicates that the odds of having severe/extreme health state among females was nearly six-fold of that among males (p-value<0.001). Similarly, the odds of having severe/extreme health state among patients with RBG >300 mg/dl and RBG in between 200–300 mg/dl were nearly threefold (p-value = 0.001) and twofold (p-value = 0.076), respectively, compared to that among patients with RBG<200 mg/dl. In addition, patients with more than one diabetes-related complications (adjusted OR = 3.5) and patients with monthly income between 5000 to 10000 (adjusted OR = 0.13) also showed a significant association with the health states.

**Table 3:** Predicators of severe/extreme health status—Adjusted odds ratio (AOR) and its 95% confidence interval.

Factors	Odds ratio (95% CI)	p-value
Gender		
Male	Reference	
Female	5.58 (2.78–11.2)	<0.001
Monthly income (in SAR)		
Less than 5000	1.80 (0.89–3.64)	0.104
5000 to 10000	0.13 (0.04–0.42)	0.001
More than 10000	Reference	
Number of diabetes related complications		
Nil	Reference	
One	2.24 (0.78–6.45)	0.136
More than one	3.54 (1.32–9.50)	0.012
Type of anti-diabetic medication		
Oral medication only	Reference	
Insulin injection or combination	1.12 (0.46–2.72)	0.806
Random blood glucose level		
less than 200	Reference	
200 to 299	3.05 (1.55–6.00)	0.001
More than 300	2.18 (0.92–5.13)	0.076

### Predictors of EQ-5D index score: Continuous modeling

Results from a multiple linear regression, where the dependent variable was the cubic function of EQ-5D index score, is presented in Table 4. Predictor variables in the logistic regression model were included. Table 4 reports the adjusted regression coefficient and its 95% confidence interval. The results confirm the findings from the logistic model that male gender, monthly income greater than 5000 SAR, having no diabetes-related complications and having RBG less than 200 mg/dl were prone to have a higher EQ-5D index compared to the corresponding contrary groups.

**Table 4:** Summary of multiple linear regression model for cubic function of EQ-5D index.

Factors	Estimate (95% CI)	p-value
Gender		
Male	Reference	
Female	-0.19 (-0.24, -0.13)	<0.001
Monthly income (in SAR)		
Less than 5000	Reference	
5000 to 10000	0.10 (0.03, 0.16)	0.004
More than 10000	0.17 (0.1, 0.24)	<0.001
Number of diabetes related complications		
Nil	Reference	
One	-0.20 (-0.27, -0.12)	<0.001
More than one	-0.10 (-0.17, -0.02)	0.013
Random blood glucose level		
less than 200	Reference	
200 to 299	-0.24 (-0.33, -0.15)	<0.001
More than 300	-0.18 (-0.24, -0.12)	<0.001

## Discussion

The burden of T2DM in Saudi Arabia is steadily increasing due to population growth, urbanization, lack of physical activity and unhealthy diet [4,25,26]. HRQoL is one of the important outcomes used to evaluate the effect of management of chronic diseases on health, and it reflects a patient’s physical and psychosocial disease burden. The present study used EQ-5D-5L to measure the HRQoL for the first time in the Arab region. Previous studies support the use of EQ-5D-5L over EQ-5D-3L as the scale with five levels has more discriminative power than the scale with three levels in patients with T2DM [27]. The present study showed moderate HRQoL with the median EQ-5D index score of 0.808. A similar finding was reported by an earlier study conducted in the Riyadh region, Saudi Arabia with a mean EQ-5D index of 0.70 [11,28]. The difference in the index score may be due to the choice of the number of levels in EQ-5D, the selection of participants to study, the quality of diabetes care, or the availability of access to support services. Another two studies conducted in Riyadh and Makah regions, Saudi Arabia, but used different measurements scales, also affirmed our finding [12,13]. A recent study from Jordan reported a similar mean EQ-5D index of 0.724 in T2DM patients in Jordan [14]. Our estimate is also consistent with findings from neighboring Middle Eastern countries [15,16]. Even though our study identified an overall moderate HRQoL, 79% of patients still had imperfect health state on some EQ-5D domains and 28% of patients reported a severe-extreme health state. Specifically, only 31% and 43% of patients expressed no problem in terms of pain/discomfort and mobility respectively. Hence, it is important to assess the influencing factors of HRQoL in patients with T2DM for the better planning of interventions to improve the physical and psychosocial burden of the disease, and hence to attain better HRQoL.

Previous studies have reported a lower HRQoL among female with diabetes compared to male with diabetes [11,12,14,29–33]. The present study also reported that HRQoL is gendered in favor of male patients with T2DM. The multivariate analysis indicated female gender as an independent predictor of poor HRQoL. The adjusted odds ratio indicates that the odds of having severe/extreme health state among females was 5.5 times higher than that among males. The multiple linear regression also confirms a higher EQ-5D index score among male patients compared to female patients. A recent systematic review reported a substantial difference in the level of physical activity favoring men in the Arab countries [34]. In addition, the socio-cultural differences between men and women in the Arab world could be a reason for the gender difference in the HRQoL. Therefore, identifying strategies to improve the quality of life among patients with diabetes, especially among women, is of great importance.

Aging has been identified as a key factor for T2DM [4,25,26] and impaired HRQoL [12,15,35,36]. Therefore, it is expected a negative association between age and HRQoL among patients with diabetes. In the present study, 30% of older patients with T2DM reported severe-extreme impaired HRQoL compared to 16% among patients aged less than 50 years. The result was not statistically significant in the regression models, which may be due to the fewer representation of younger patients in our study sample.

Studies have demonstrated that socioeconomic status is positively associated with HRQoL among adults with a chronic disease [37]. In the present study, a higher proportion (40%) of patients having primary education or lower reported severe-extreme impaired HRQoL compared to patients having higher education; which is consistent with a previous study from Oman [15]. Similarly, a higher proportion (42%) of patients with low monthly income reported severe-extreme impaired HRQoL compared to patients having moderate/high monthly income. However, the multiple regression models did not find a significant difference in EQ-5D index between the educational levels. As Robert et al pointed out in a study, an improvement in HRQoL of people at the lowest end of the socioeconomic distribution helps substantial improvement in the HRQoL at the population level [38].

The present study reports a higher proportion, but not statistically significant in the logistic regression model, of patients with severe-extreme impaired HRQoL among patients under insulin therapy compared to the contrary group. The difference in EQ-5D index score was also not significant based on the multiple regression model. HRQoL can be positively and negatively associated with insulin therapy [39]. Due to the beneficial effects of the insulin therapy, such as better glycemic control and lower risk of diabetic complications, better rating may have been given on domains of HRQoL [40]. Conversely, the inconvenience associated with the insulin therapy, fear of weight gain and the risk of hypoglycemia may adversely affect the patient’s HRQoL [40]. In the current study, having multiple complications of diabetes was found to be negatively associated with HRQoL. Patients who had more than one complications reported lower EQ-5D score, in consistent with other studies that explored the relationship [12].

Many studies have previously reported that the severity of T2DM has a negative impact on quality of life [41,42], however, the impact of the level of RBG on HRQoL is still uncertain. Kayo et al reported RBG level was negatively associated with cognitive impairment in the elderly [43]. A recent study reported a non-significant relationship between quality of life with glucose levels among Iranian diabetic patients [44]. However, our study showed a strong negative association between random glucose level and HRQoL. The adjusted odds ratios indicate that the odds of having severe-extreme health state among patients with RBG >200 mg/dl was more than twofold of that among patients with RBG<200 mg/dl. In addition, the multiple linear regression also confirmed a significant reduction in EQ-5D index score against an increase in RBG level. A high RBG usually account for the poor control of diabetes, and hence it may negatively affect the HRQoL.

Some limitations should be noted. The study was of a cross-sectional design and thus the association that has been demonstrated in our study may not imply a causal relationship. Importantly, the study was restricted to patients from two outpatient health centers of a major tertiary hospital in Eastern Province, Saudi Arabia. However, a study on diabetes-related QoL has not been reported at the national level or from Eastern Province in the past decade, and hence the importance of our study. Although the study was restricted to patients with a minimum one-year duration of diabetes, the actual duration was not obtained. HbA1c, which may be a better indicator for glucose control than random glucose level, was also not collected.

## Conclusion

This study demonstrates a moderate HRQoL among patients with T2DM. The impaired HRQoL is mainly in terms of pain/discomfort and mobility due to diabetes. The results showed that male gender, high income, without complications and good glucose control have relatively better quality of life. The study will help in guiding the development of effective intervention programs to improve T2DM related HRQoL among the Saudi population. Such programs should target especially at groups with female gender, older age, low socio-economic status, multiple complications of diabetes and high RBG.

## References

[1] International Diabetes Federation. IDF Diabetes Atlas, 8th edn Brussels, Belgium: 2017.

[2] Zheng Y, Ley SH, Hu FB. Global aetiology and epidemiology of type 2 diabetes mellitus and its complications. Nat Rev Endocrinol 2017;14:88–98. 10.1038/nrendo.2017.151 29219149

[3] World Health Organization Global Health Estimates 2016: Disease burden by Cause, Age, Sex, by Country and by Region, 2000–2016. Geneva: World Health Organization; 2018.

[4] Alneami YM, Coleman CL. Risk Factors for and Barriers to Control Type-2 Diabetes among Saudi Population. Glob J Health Sci 2016;8:54089 10.5539/gjhs.v8n9p10 27157156 PMC5064063

[5] Robert A, Al Dawish M, Braham R, Musallam M, Al Hayek A, Al Kahtany N. Type 2 Diabetes Mellitus in Saudi Arabia: Major Challenges and Possible Solutions. Curr Diabetes Rev 2016;13:59–64. 10.2174/1573399812666160126142605 26813972

[6] World Health Organization. Global report on diabetes. Geneva: World Health Organization; 2017.

[7] Wang H, Naghavi M, Allen C, Barber RM, Bhutta ZA, Carter A, et al Global, regional, and national life expectancy, all-cause mortality, and cause-specific mortality for 249 causes of death, 1980–2015: a systematic analysis for the Global Burden of Disease Study 2015. Lancet 2016;388:1459–544. 10.1016/S0140-6736(16)31012-1 27733281 PMC5388903

[8] Fitzpatrick R, Fletcher A, Gore S, Jones D, Spiegelhalter D, Cox D. Quality of life measures in health care. I: Applications and issues in assessment. BMJ 1992;305:1074–7. 10.1136/bmj.305.6861.1074 1467690 PMC1883623

[9] van der Vinne E. The ultimate goal of disease management: improved quality of life by patient centric care. Int J Integr Care 2009;9:e89 10.5334/ijic.321 19777113 PMC2748182

[10] Mokdad AH, Tuffaha M, Hanlon M, El Bcheraoui C, Daoud F, Al Saeedi M, et al Cost of Diabetes in the Kingdom of Saudi Arabia. J Diabetes Metab 2015;6:575.

[11] Al-Aboudi IS, Hassali MA, Shafie AA, Hassan A, Alrasheedy AA. A cross-sectional assessment of health-related quality of life among type 2 diabetes patients in Riyadh, Saudi Arabia. SAGE Open Med 2015;3:2050312115610129 10.1177/2050312115610129 26770806 PMC4679330

[12] Al Hayek AA, Robert AA, Al Saeed A, Alzaid AA, Al Sabaan FS. Factors Associated with Health-Related Quality of Life among Saudi Patients with Type 2 Diabetes Mellitus: A Cross-Sectional Survey. Diabetes Metab J 2014;38:220 10.4093/dmj.2014.38.3.220 25003076 PMC4083029

[13] Elazhary H, Noorwali A, Zaidi N, Alshamrani R, Aljohani M, Khan D, et al Knowledge about Diabetes and Its Effect on Quality of Life among Diabetic Patients in King Abdulaziz University Hospital, Jeddah. J Adv Med Med Res 2018;26:1–11. 10.9734/JAMMR/2018/40083

[14] Jarab AS, Alefishat E, Mukattash TL, Albawab AQ, Abu-Farha RK, McElnay JC. Exploring variables associated with poor health-related quality of life in patients with type 2 diabetes in Jordan. J Pharm Heal Serv Res 2018 10.1111/jphs.12255

[15] Al-Maskari MY, Al-Shookri AO, Al-Adawi SH, Lin KG, Al-Shookri A. Assessment of quality of life in patients with type 2 diabetes mellitus in Oman. Saudi Med J 2011;32:1285–90. 22159385

[16] Bani-Issa W. Evaluation of the health-related quality of life of Emirati people with diabetes: integration of sociodemographic and disease-related variables. East Mediterr Heal J 2011;17:825–30. 10.26719/2011.17.11.825 22276489

[17] Trikkalinou A, Papazafiropoulou AK, Melidonis A. Type 2 diabetes and quality of life. World J Diabetes 2017;8:120–9. 10.4239/wjd.v8.i4.120 28465788 PMC5394731

[18] Safita N, Islam SMS, Chow CK, Niessen L, Lechner A, Holle R, et al The impact of type 2 diabetes on health related quality of life in Bangladesh: results from a matched study comparing treated cases with non-diabetic controls. Health Qual Life Outcomes 2016;14:129 10.1186/s12955-016-0530-7 27624600 PMC5022158

[19] Sakamaki H, Ikeda S, Ikegami N, Uchigata Y, Iwamoto Y, Origasa H, et al Measurement of HRQL Using EQ-5D in Patients with Type 2 Diabetes Mellitus in Japan. Value Heal 2006;9:47–53. 10.1111/j.1524-4733.2006.00080.x 16441524

[20] EuroQol Group. EuroQol—a new facility for the measurement of health-related quality of life. Health Policy 1990;16:199–208. 10.1016/0168-8510(90)90421-9 10109801

[21] Devlin NJ, Shah KK, Feng Y, Mulhern B, van Hout B. Valuing health-related quality of life: An EQ-5D-5L value set for England. Health Econ 2018;27:7–22. 10.1002/hec.3564 28833869 PMC6680214

[22] Bekairy AM, Bustami RT, Almotairi M, Jarab A, Katheri AM, Aldebasi TM, et al Validity and reliability of the Arabic version of the the EuroQOL (EQ-5D). A study from Saudi Arabia. Int J Health Sci (Qassim) 2018;12:16–20. 29599689 PMC5870320

[23] Aburuz S, Bulatova N, Twalbeh M, Gazawi M. The validity and reliability of the Arabic version of the EQ-5D: a study from Jordan. Ann Saudi Med 2009;29:304–8. 10.4103/0256-4947.55313 19584581 PMC2841459

[24] Herdman M, Gudex C, Lloyd A, Janssen M, Kind P, Parkin D, et al Development and preliminary testing of the new five-level version of EQ-5D (EQ-5D-5L). Qual Life Res 2011;20:1727–36. 10.1007/s11136-011-9903-x 21479777 PMC3220807

[25] Moradi-Lakeh M, Forouzanfar MH, El Bcheraoui C, Daoud F, Afshin A, Hanson SW, et al High Fasting Plasma Glucose, Diabetes, and Its Risk Factors in the Eastern Mediterranean Region, 1990–2013: Findings From the Global Burden of Disease Study 2013. Diabetes Care 2017;40:22–9. 10.2337/dc16-1075 27797926

[26] Sherif S, Sumpio BE. Economic development and diabetes prevalence in MENA countries: Egypt and Saudi Arabia comparison. World J Diabetes 2015;6:304–11. 10.4239/wjd.v6.i2.304 25789111 PMC4360423

[27] Pan C-W, Sun H-P, Wang X, Ma Q, Xu Y, Luo N, et al The EQ-5D-5L index score is more discriminative than the EQ-5D-3L index score in diabetes patients. Qual Life Res 2015;24:1767–74. 10.1007/s11136-014-0902-6 25540029

[28] Al-Aboudi IS, Hassali MA, Shafie AA. Knowledge, attitudes, and quality of life of type 2 diabetes patients in Riyadh, Saudi Arabia. J Pharm Bioallied Sci 2016;8:195–202. 10.4103/0975-7406.171683 27413347 PMC4929958

[29] Al-Shehri AH, Taha AZ, Bahnassy AA, Salah M. Health-related quality of life in type 2 diabetic patients. Ann Saudi Med 2008;28:352–60. 10.5144/0256-4947.2008.352 18779640 PMC6074492

[30] Quah JHM, Luo N, Ng WY, How CH, Tay EG. Health-related quality of life is associated with diabetic complications, but not with short-term diabetic control in primary care. Ann Acad Med Singapore 2011;40:276–86. 21779616

[31] Redekop WK, Koopmanschap MA, Stolk RP, Rutten GEHM, Wolffenbuttel BHR, Niessen LW. ealth-related quality of life and treatment satisfaction in Dutch patients with type 2 diabetes. Diabetes Care 2002;25:458–63. 10.2337/diacare.25.3.458 11874930

[32] Lee WJ, Song K-H, Noh JH, Choi YJ, Jo M-W. Health-Related Quality of Life Using the EuroQol 5D Questionnaire in Korean Patients with Type 2 Diabetes. J Korean Med Sci 2012;27:255 10.3346/jkms.2012.27.3.255 22379335 PMC3286771

[33] Unden A, Elofsson S, Andreasson A, Hillered E, Eriksson I, Brismar K. Gender differences in self-rated health, quality of life, quality of care, and metabolic control in patients with diabetes. Gend Med 2008;5:162–80. 10.1016/j.genm.2008.05.003 18573483

[34] Sharara E, Akik C, Ghattas H, Makhlouf Obermeyer C. Physical inactivity, gender and culture in Arab countries: a systematic assessment of the literature. BMC Public Health 2018;18:639 10.1186/s12889-018-5472-z 29776343 PMC5960209

[35] Al Senany S, Al Saif A. Assessment of physical health status and quality of life among Saudi older adults. J Phys Ther Sci 2015;27:1691–5. 10.1589/jpts.27.1691 26180299 PMC4499962

[36] Khoja AT, Aljawadi MH, Al-Shammari SA, Mohamed AG, Al-Manaa HA, Morlock L, et al The health of Saudi older adults; results from the Saudi National Survey for Elderly Health (SNSEH) 2006–2015. Saudi Pharm J 2018;26:292–300. 10.1016/j.jsps.2017.11.008 30166931 PMC6111452

[37] Mielck A, Vogelmann M, Leidl R. Health-related quality of life and socioeconomic status: inequalities among adults with a chronic disease. Health Qual Life Outcomes 2014;12:58 10.1186/1477-7525-12-58 24761773 PMC4011770

[38] Robert SA, Cherepanov D, Palta M, Dunham NC, Feeny D, Fryback DG. Socioeconomic Status and Age Variations in Health-Related Quality of Life: Results From the National Health Measurement Study. Journals Gerontol Ser B Psychol Sci Soc Sci 2009;64B:378–89. 10.1093/geronb/gbp012 19307286 PMC2670253

[39] Ishii H, Terauchi Y, Jinnouchi H, Taketsuna M, Takeuchi M, Imaoka T. Effects of insulin changes on quality of life and glycemic control in Japanese patients with type 2 diabetes mellitus: The insulin-changing study intending to gain patients’ insights into insulin treatment with patient-reported health outcomes in actual c. J Diabetes Investig 2013;4:560–70. 10.1111/jdi.12086 PMC4020251 24843710

[40] Home P, Riddle M, Cefalu WT, Bailey CJ, Bretzel RG, Del Prato S, et al Insulin therapy in people with type 2 diabetes: opportunities and challenges? Diabetes Care 2014;37:1499–508. 10.2337/dc13-2743 24855154 PMC5131884

[41] Navicharern R. Diabetes self-management, fasting blood sugar and quality of life among type 2 diabetic patients with foot ulcers. J Med Assoc Thai 2012;95:156–62. 22435243

[42] Scollan-Koliopoulos M, Bleich D, Rapp KJ, Wong P, Hofmann CJ, Raghuwanshi M. Health-Related Quality of Life, Disease Severity, and Anticipated Trajectory of Diabetes. Diabetes Educ 2013;39:83–91. 10.1177/0145721712467697 23174664

[43] Kayo AR, Wimala AR, Angela N, Rashid IA. Random blood glucose level as predictor of cognitive impairment in elderly. Univ Med 2012;31:131–9.

[44] Parsa P, Ahmadinia-Tabesh R, Mohammadi Y, Khorami N. Investigating the relationship between quality of life with lipid and glucose levels in Iranian diabetic patients. Diabetes Metab Syndr Clin Res Rev 2017;11:S879–83. 10.1016/J.DSX.2017.07.009 28755844

