# Supplementary material for: Health-related quality of life among patients with type 2 diabetes mellitus in Eastern Province, Saudi Arabia: A cross-sectional study
Source: PLoS One. 2020 Jan 10;15(1):e0227573. doi: 10.1371/journal.pone.0227573 (PMC6953887; doi:10.1371/journal.pone.0227573)
Supplement: Attachment — Submitted filename: Response to Reveiwers.docx [file pone.0227573.s001.docx]

**Response to academic editor**

1. Please ensure that your manuscript meets PLOS ONE's style requirements, including those for file naming. The PLOS ONE style templates can be found at <http://www.plosone.org/attachments/PLOSOne_formatting_sample_main_body.pdf> and <http://www.plosone.org/attachments/PLOSOne_formatting_sample_title_authors_affiliations.pdf>

**The manuscript has been modified according to the journal’s style requirement (Refer page 1, L2-3).**

2. Please refer to any sample size calculation performed prior to participant recruitment. If this was not performed please justify the reasons. Please refer to our statistical reporting guidelines for assistance (<https://journals.plos.org/plosone/s/submission-guidelines.#loc-statistical-reporting>).

**Details on sample size calculation has been added to the methods section (Refer P 5; L90-93). “A minimum sample size of 385 was calculated by assuming 50% of patients were adherent to treatments with the absolute precision of 0.05 and 95% confidence level. The 50% was purposively selected so that it provided the largest minimum sample size.”**

3. Your ethics statement must appear in the Methods section of your manuscript. If your ethics statement is written in any section besides the Methods, please move it to the Methods section and delete it from any other section. Please also ensure that your ethics statement is included in your manuscript, as the ethics section of your online submission will not be published alongside your manuscript.

**Ethics statement was included in the methods section (Page 5, L100-101).**

4. We note that you have indicated that data from this study are available upon request. PLOS only allows data to be available upon request if there are legal or ethical restrictions on sharing data publicly. For information on unacceptable data access restrictions, please see <http://journals.plos.org/plosone/s/data-availability#loc-unacceptable-data-access-restrictions>. In your revised cover letter, please address the following prompts:

**We have addressed the following in the revised cover letter.**

**“We have received consent from the participants for participation in the research and publication of results. However, we were not consented for sharing the data publicly by the participants. For queries related to the study data, Dr. Mohamed Baraka, member of IRB, IAU can be contacted on his email:** [**mabaraka@iau.edu.sa**](mailto:mabaraka@iau.edu.sa) (IRB approval number: IRB-2109-05-391).**”**

**Response to reviewers:**

Reviewer #1:

1. In the title the word deterioration gives you the sense that you follow healthy participants and then you study deterioration for a period of time but in this study the main objective is to assess the quality of life and to look for predictors, so my suggestion is to change the title to suit with the objectives.

**We thank the reviewer for the valid point. We have modified the title accordingly. The title is “Health-related quality of life among patients with type 2 diabetes mellitus in Eastern Province, Saudi Arabia: a cross-sectional study”**

2. Usually the use of abbreviations is not suitable in the abstract unless you clarify them even international abbreviations.

**Corrected (Page 2)**

3. The methodology needs to be revised; you have to mention how you determine the amount of sample and why you select two centers and how many centers in total in the area, all these things should be mentioned.

**We have revised the methodology section by including details of sample size calculation (Page 5, L94-97) and sampling methodology (Page 5, L89-94).**

**We calculated a minimum sample size of 385 assuming 50% of patients were adherent to treatments with the absolute precision of 0.05 and 95% confidence level. The 50% was purposively selected so that it provided the largest minimum sample size. As mentioned in the methodology section, we considered two health centres in the Eastern Province, Saudi Arabia out of five state owned general hospitals in the province. These two centres, which are affiliated with a largest university in the region, serves larger volume of patients from different geographical locations in the region compared to other hospitals.**

Reviewer #2:

1. According to my knowledge, it is a novel paper in its field opening new horizons for further evidence. In addition, the object as well as the results are appropriately discussed in the context of previous literature explaining the importance of the manuscript in its field. Authors succeed to present their data in a clear way adding information to the existing literature.
   Therefore, I have no corrections or further work to propose for the improvement of the manuscript and therefore it can be published unaltered.

**We thank the reviewer for his good feedback.**
